# A Flow-Through Cell Electroporation Device for Rapidly and Efficiently Transfecting Massive Amounts of Cells *in vitro* and *ex vivo*

**DOI:** 10.1038/srep18469

**Published:** 2016-01-05

**Authors:** Deyao Zhao, Dong Huang, Yang Li, Mengxi Wu, Wenfeng Zhong, Qiang Cheng, Xiaoxia Wang, Yidi Wu, Xiao Zhou, Zewen Wei, Zhihong Li, Zicai Liang

**Affiliations:** 1 Institute of Molecular Medicine, Peking University, Beijing 100871, China; 2 National Key Laboratory of Science and Technology on Micro/Nano Fabrication, Institute of Microelectronics, Peking University, Beijing 100871, China; 3 Department of Engineering Science and Mechanics, The Pennsylvania State University, State College, PA 16801, USA; 4 National Center for Nanoscience and Technology, Beijing 100190, China

## Abstract

Continuous cell electroporation is an appealing non-viral approach for genetically transfecting a large number of cells. Yet the traditional macro-scale devices suffer from the unsatisfactory transfection efficiency and/or cell viability due to their high voltage, while the emerging microfluidic electroporation devices is still limited by their low cell processing speed. Here we present a flow-through cell electroporation device integrating large-sized flow tube and small-spaced distributed needle electrode array. Relatively large flow tube enables high flow rate, simple flow characterization and low shear force, while well-organized needle array electrodes produce an even-distributed electric field with low voltage. Thus the difficulties for seeking the fine balance between high flow rate and low electroporation voltage were steered clear. Efficient *in vitro* electrotransfection of plasmid DNA was demonstrated in several hard-to-transfect cell lines. Furthermore, we also explored *ex vivo* electroporated mouse erythrocyte as the carrier of RNA. The strong ability of RNA loading and short exposure time of freshly isolated cells jointly ensured a high yield of valid carrier erythrocytes, which further successfully delivered RNA into targeted tissue. Both *in vitro* and *ex vivo* electrotransfection could be accomplished at high cell processing speed (20 million cells per minute) which remarkably outperforms previous devices.

Electroporation has found to be a promising non-viral physical technology at the cellular level for the delivery of various molecules^1^,^2^,^3^, including oligo DNA, interference RNA and molecular drugs. Since the first commercial electroporation device was released in 1990s, the cuvette-like bulk electroporation devices have been wildly employed as a research tool. Unfortunately, the cell processing speed of bulk electroporation devices was limited due to the discontinuous operation. Typically, it costs around 5 minutes to process one batch of cells (about 5 × 10^5^ cells). Therefore, the bulk electroporation devices are inadequate for many biological studies, such as drug screening, antibody production and molecular therapy, in which a large amount of cells need to be transfected rapidly^4^. For example, in tumor immune therapy, 10^8^ ~ 10^9^ immune cells need to be *ex vivo* transfected and re-transfused to patient in few hours^5^. To address the issue of cell processing speed, the continuous cell electroporation was firstly demonstrated by proof-of-concept devices^6^,^7^, in which two tubes were directly assembled on two opposite side walls of a cuvette. Since then, a number of studies^8^,^9^,^10^,^11^ have been undertaken to increase the cell processing speed and improve the transfection efficiency and/or the cell viability. However, for such devices employing plate-like electrodes with relatively large spacing (several millimetres to centimetres), the transfection efficiency and cell viability remained unsatisfactory, mainly due to multiple harmful effects induced by high electroporation voltage. Utilizing the microfluidic technology^12^,^13^,^14^,^15^, the spacing between electrodes could be shrunk to a few tens of microns, and the electroporation voltage was accordingly reduced to a few volts. In addition, the microfabrication also enabled the precisely optimization of the channel and/or electrode geometries, along with the possibility of integrating different functional unit^16^, such as cell pumping and plasmid mixing^17^,^18^. Therefore, microfluidic electroporation devices exhibited better transfection efficiency and cell viability than macro-scale devices^4^,^19^. However, the cell processing speed of microfluidic devices was limited by the small volume of the channel and the restricted flow velocity. To the best of our knowledge, the existing microfluidic electroporation devices could only process less than millions cells, which is insufficient for many practical applications, such as molecular therapy. Overall, the macro-scale continuous systems ensured the high cell processing speeds, yet suffered from the adverse effects caused by their high voltage. Contrarily, the microfluidic devices improved the transfection efficiency and cell viability by precisely controlling the geometric size of both electrodes and flow channel, yet sacrificed the cell processing speed due to the limited cross-sectional area of microfluidic channel.

To address these issues, this study explored a different strategy. We integrated a macro-scale flow channel and a micro-scale electrode array together to ensure the high cell processing speed and the fine electroporation performance simultaneously. A relatively big cylinder-shaped glass tube (inner diameter 6.8 mm) was employed as the flow channel to enable high flow rate, simple flow characterization and low shear force, while 37 pillared electrodes were carefully arranged as a cellular hexagonal array, producing an even-distributed electric field. Also, by realizing that the adverse effects occurred around the cathode compromised the cell viability, a tri-phase electrical stimulation mode was introduced to alleviate these harmful effects, including heat accumulation and pH value change. After optimizing the electrical and hydrodynamic parameters, we achieved high nucleic acid transfection efficiency (up to 60%) and fine cell viability (up to 80%) on various cell lines. Furthermore, we successfully delivered RNA into freshly isolated mouse erythrocyte and re-transfused the erythrocyte back as the RNA carrier. The RNA was efficiently released into kidney and spleen. In this study, the flow-through electroporation device maintained a high processing speed (2.25 × 10^7^ cells per minute).

## Experimental Section

### Materials

A GFP (pEGFP-C3) plasmid which encodes green fluorescence protein was used to determine the DNA transfection efficiency of electroporation *in vitro*. A commercial kit (EndoFree Plasmid Maxi Kit, TIANGEN, China) was employed to purify GFP plasmid. A Cy5-labelled RNA (Ribo Co., China) was used to determine the RNA delivery efficiency of electroporation *ex vivo*. The sequence of RNA is:

sense: 5′-Cy5-CCUUGAGGCAUACUUCAAAdTdT-3′;

antisense: 5′-UUUGAAGUAUGCCUCAAGGdTdT-3′.

It was stablized with certain chemical modifications and with a Cy5 fluorophore on the 5′ of the sense strand. A modified hypo-osmolar electroporation buffer (25 mM KCl, 0.3 mM KH_2_PO_4_, 0.85 mM K_2_HPO_4_, 36 mM myo-inositol) was used in *in vitro* electroporation assays. For ex vivo electroporation, the myo-inositol was adjusted to 126 mM to achieve a suitable osmotic pressure for mouse erythrocyte.

### Cells and animals preparation

For *in vitro* electroporation, HEK-293A cells were grown in Dulbecco’s modified Eagle’s medium (DMEM), HL-60 and CCRF-CEM cells were grown in RPMI-1640 medium. Both kinds of media were supplemented with 10% fetal bovine serum (Sigma), 100 units/ml penicillin and 100 μg/ml streptomycin (Gibico). Cells were incubated at 37°C in 5% CO_2_ humidified atmosphere. All cells were seeded in a culture flask (Corning) 2–3 days prior to the experiments. For *ex vivo* electroporation, Female C57BL/6 mice (age 6–8 weeks) were purchased from Vital River Laboratories (Beijing, China). Animals were maintained in Peking University Laboratory Animal Center, which is an AAALAC-accredited and specific pathogen free (SPF) experimental animal facility. All of the experimental animals in our study were treated in accordance with protocols approved by the Institutional Animal Care and Use Committee of Peking University.

### Experiment protocols and determination of the nucleic acid transfection efficiency

For *in vitro* electroporation, cultured cells were harvested by trypsin treatment and resuspended to a density of about 5 × 10^6^ cells/mL in the hypo-osmolar buffer. Then GFP plasmid was added to a final concentration of 20 μg/mL. Using an infusion pump (Terumo Co. STC 503, Japan), the mixture was pumped into the electroporation device through the inlet. The flow speed was adjusted according demands. The needle electrode array was connected to a pulse generator (BTX Apparatus, ECM 830, USA), generating required electrical field in the electroporation chamber. The cells were electroporated while pass the electroporation chamber. The electrical parameters were individually optimized for different cell types. After being electroporated, cells were collected from the outlet and cultured. 24 hours later, the number of GFP-expressing cells was counted in five randomly chosen fields, using ImageJ from NIH, under a fluorescence microscope (Olympus Co., IX71, Japan). The fluorescence threshold of GFP-expressing cells was manual determined by visually checking the transfected cells. For each transfection, transfection rate (TR) was calculated by dividing the number of GFP-expressing cells by the total cell number.

For *ex vivo* electroporation, 500 μL blood was collected from each mouse through orbit and then washed three times by 0.9% NaCl. After discarding the supernatant, 60 μL blood plasma was taken and mixed with 240 μL electroporation buffer and 30 μL Cy-5 labelled RNA. The mixture was firstly preloaded into a syringe and then pumped into the electroporation chamber through the inlet. After electroporation, the mixture was collected through the outlet and wash three times by 0.9% NaCl to eliminate possible cell debris and residual RNA which remained outside of erythrocytes. The washed mixture was fluorescently imaged using an In-Vivo Imaging System (Carestream In-Vivo Imaging System FX Pro, Carestream Health, USA) to determine the fluorescence intensity which represented the amount of Cy-5 labelled RNA inside the erythrocytes. To evaluate the RNA releasing effect, the electroporated mixture was resuspended in 500 μL PBS (Phosphate Buffered Saline) solution and transfused back to a mouse through tail intravenous injection. Six hours later, the mouse was sacrificed and the fluorescence intensities of several internal organs were quantitatively detected by the In-Vivo Imaging System. Using student’s t-test, the significance of the fluorescence intensities was analyzed.

## Results and Discussions

### A flow-through cell electroporation device

**Figure 1:**
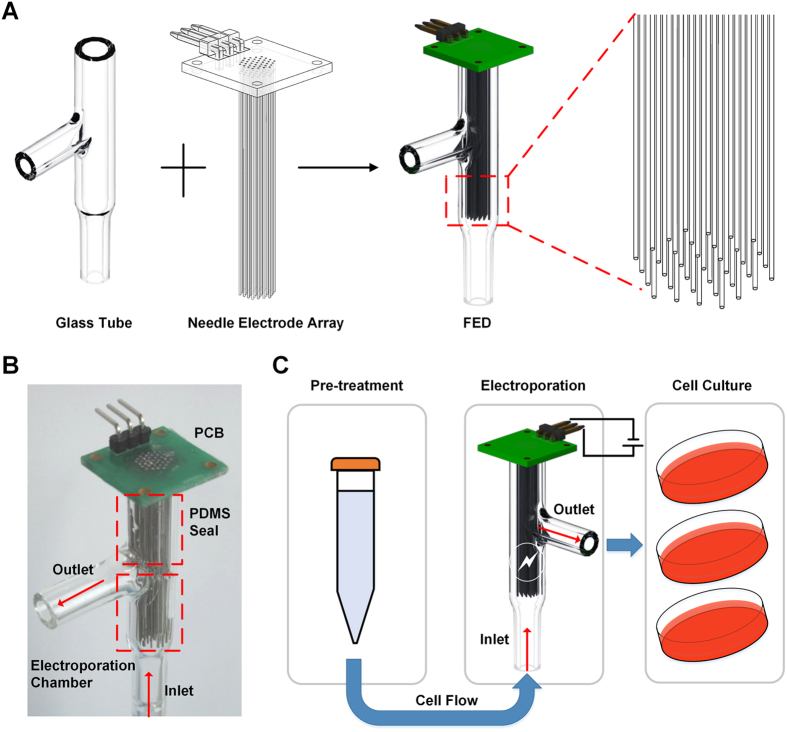
The flow-through cell electroporation devices (FED) and its operation process. (**A**)The schematic of the FED structure. The FED consists of two components, a glass tube and a needle electrode array (NEA) welded on a PCB. In the assembled FED, the needle-like electrodes are evenly distributed in the electroporation chamber. The close-up view of NEA shows its arrangement. (**B**) The photos of FED, the PDMS seal area and the electroporation chamber are labelled by red dotted boxes, respectively. (**C**) The operation process of FED includes three procedures: (i) The cells are firstly pre-treated, including being washed and mixed with electroporation buffer and nucleic acid. (ii) Then the cells are pumped into the FED through the inlet and electroporated in the FED. The NEA is connected to a pulse generator through the specially-designed PCB. g (iii) The electroporated cells are collected for culture through the outlet. The cell flow direction is indicated by red arrows.

As shown in Fig. 1A, the flow-through cell electroporation device (FED) consisted of two components, a T-shaped glass tube with three ends and a needle-electrode array (NEA). The glass tube, which served as the cell flow channel, was purposely designed to have a relatively large inner diameter (6.8 mm). The cross-sectional area of the glass tube was about 36 mm^2^, which was approximately 360 times larger than the typical cross-sectional area (0.1 mm^2^) of the representative micro-fluidic electroporation devices^15^. On the other hand, every flowing cell experiences a shear force which is proportional to the flow velocity, and the shear force must be under a certain limit, or the cell viability will be severely harmed. For this reason, each kind of cell is supposed to have a fixed upper limit of its flow velocity, no matter in microfluidic channel or in glass tube. Therefore, with the same flow velocity, the glass tube could process the cell suspension hundreds times faster than microfluidic devices could do. The bottom end and the side end of the glass tube severed as the cell inlet and outlet, respectively, while the top end was utilized as the assemble port for the NEA. The NEA consisted of 37 needle-shaped electrodes which were carefully arranged as a cellular hexagonal array to generate an even-distributed electrical field in the glass tube for cell electroporation. All needle electrodes were welded a PCB (printed circuit board) to achieve controlled electrical connection. After assembling the NEA into the glass tube, the upper part of the glass was sealed with polydimethylsiloxane (PDMS, Dow Corning, USA) for the electrical isolation between cell suspension and PCB. Figure 1B shows the finished FED, in which the inlet, the outlet and the electroporation chamber was marked. The length of the effective electroporation chamber was 1.5 cm. As schemed in Fig. 1C, the cells were firstly pre-treated (described in section 2.3), then continuously pumped into the electroporation chamber through the inlet. After being electroporated while passing the chamber, the electrotransfected cells were accordingly collected through the outlet for culture. The detailed descriptions of the device setup and the operation procedure were included in the Supplementary Figure S1.

**Figure 2:**
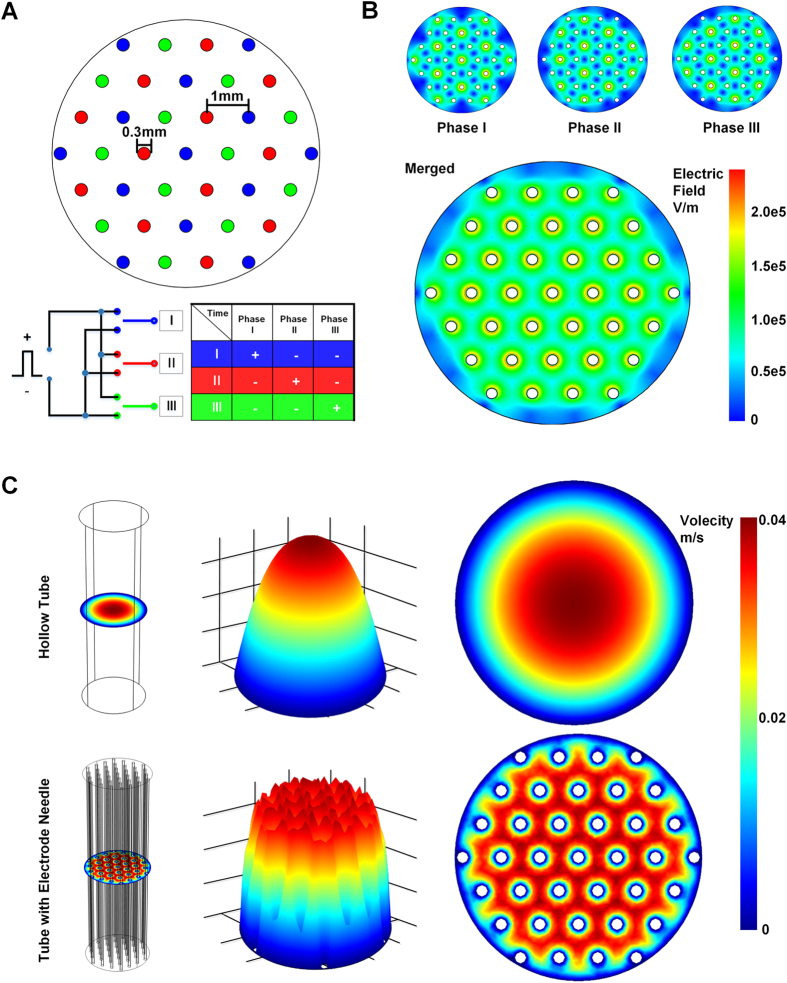
The arrangement of the needle electrode array (NEA) and the simulations of electrical field and flow velocity distribution. (**A**) The arrangement of the NEA and schematic diagram of tri-phase electrical stimulation mode. Electrodes colored in blue, red and green are connected to pole I, II and III respectively, and electric polarity is alternated during three phases. (**B**) Simulation of electric field distribution during three phases and the equivalent field intensity. The lower image shows the equivalent electric field if three phases are merged. (**C**) Simulations of the flow velocity distribution inside the glass tube. The upper row shows the flow field inside a glass tube without NEA. The lower row shows the flow field inside a glass tube with NEA. For both rows, the left image indicates the model setup and the zero XY plane of the simulation; the middle image shows the 3-dimension distribution of the flow velocity; the right image is the flow velocity distribution on the zero XY plane.

Distinguished from previous cuvette-like electroporation devices which placed two electrodes on opposite side walls of the cuvette, our FED employed 37 needle electrodes as a distributed network and placed the electrodes directly in the electroporation chamber, benefiting the device performance both electrically and hydro-dynamically. Figure 2A demonstrates the arrangement and electrical connection of the NEA. The diameter of each electrode was 0.3 mm while the spacing between neighbouring electrodes was 0.7 mm. The electrodes were divided into three groups, as colored in blue, red and green. Each group was connected to a circuit consisted of three switches. The circuit sensed every electric pulse, and then alternated the switches to shift the polarity. Therefore, for each pulse, only one group was connected to the anode, while other two groups were connected to the cathode. As shown in Fig. 2B, the electric field generated by such an electrode array simulated by a FEA (finite element analysis) software, COMSOL ver 3.5a. During a circulation of electrode shifting, an even distributed electric field covered most area of the flow tube area. This special arrangement of the electrodes improved the coverage of effective electric field and relieved the harmful effect occurred around the electrode. These advantages were verified by our previous study^20^. Furthermore, embedding the NEA into the glass tube also improved the uniformity of the flow velocity. As simulated in Fig. 2C, for a hollow tube, the flow velocity in the tube center is higher than the velocity around the tube rim, due to the viscous force between the fluid and the tube wall. Embedding the NEA could be considered as introducing viscous force to the fluid around each electrode, therefore averaging the flow velocity all over the tube. The gradient of flow velocity is corresponding reduced. Considering the shear force acting on a round-shaped cell with specific diameter is proportional to the gradient of flow velocity^21^. Therefore embedding the NEA helped avoiding the unnecessary cell death induced by excessive shear force and ensuring constant electroporation efficiency for all cells.

Differed from those micro-scale electroporation devices whose characteristic dimensions were as small as dozens of microns, our FED avoided the requirements of the clean-room facilities, therefore remarkably reduced the cost and enhanced the modifiability. The detailed fabrication process was described in Supplementary Figure S2. Briefly, the glass tube and PCB board were custom-made. The needle electrodes were modified from bio-compatible stainless steel acupuncture needles. The needle electrodes were manually welded on the PCB board to form the NEA. An important point is to maintain all needle electrodes in parallel, otherwise the electric filed would be uneven, or even worse, the short circuit might occur. An additional alignment board which had the same pattern with the PCB board was used to help the alignment. The finished NEA was inserted into the glass tube through the upper end to form the FED. After sealing the upper part of the glass tube with PDMS, the devices was finished.

### DNA transfection *in vitro*

**Figure 3:**
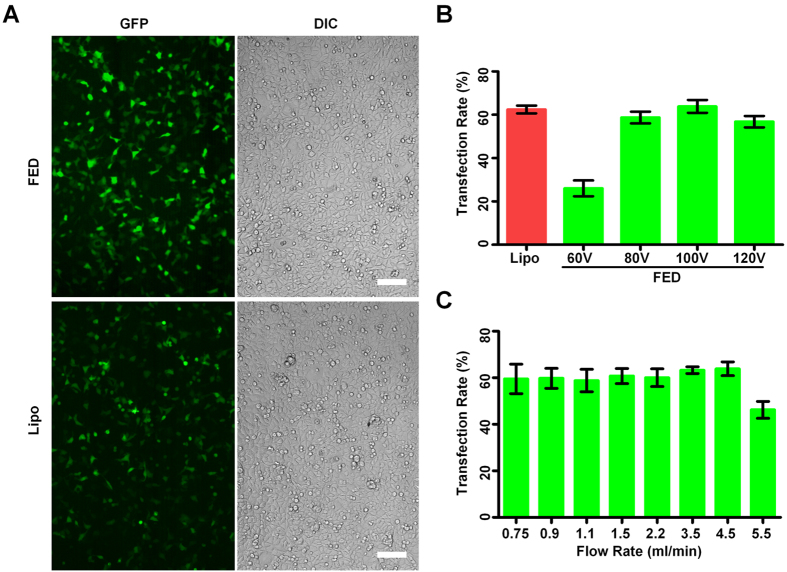
Electrotransfecting HEK-293A cells with plasmid DNA. (**A**) To determine the electroporation efficiency of the FED, pEGFP plasmid was transfected into HEK-293A cells (upper two images), while the cells transfected by Lipofectamine2000 were used for comparison (lower two images).The green spots represent transfected cells, which expressed GFP and exhibited green fluorescence. Scale bar 100 μm. (**B**) The transfection rate of Lipofectamine2000 and electroporation with different voltages. The flow rate is 4.5 ml/min. (**C**) The relationship between transfection rate and cell flow rate. The voltage is 100 V. In (**B,C**), the transfection rate is calculated by dividing the number of GFP-expressing cells by the total cell number. The cell number is counted in five randomly chosen fields of each fluorescent image, using ImageJ from NIH. Each column is the average of three independent assays, each data is showed as the mean ± S.D.

To evaluate the cell transfection performance of the FED, we used HEK-293A cells and plasmid DNA (pEGFP-C3) as model system of DNA transfection. The operating procedure is described in section 2.3. To determine whether the transfection efficiency of FED could fulfil the general requirement of biological studies, we employed a widely accepted laboratorial transfection reagents (Lipofectamine2000, Lipo) as the comparison. Figure 3A shows the fluorescent images of cells respectively transfected by FED and Lipo. It revealed that the FED and Lipo had similar DNA transfection rate (TR, transfected cells/total cells), about 60%. According to established knowledge^15^,^22^ and our previous chip electroporation studies^14^,^20^,^23^, the electrical field strength was believed to be the key factor deciding the TR. We then investigated the relationship between the electroporation voltage and the TR by quantitatively assessing the ratio of GFP transfected cells. Other electric parameters (pulse duration 0.1 ms, pulse interval 1 s) for electroporation were optimized according to our previous work^13^. Briefly, we firstly settled buffer ionic strength and osmolarity, then adjusted the pulse duration and pulse switching interval to get the best balance between transfection efficiency and cell viability. The analyzing method is described in section 2.3. Figure 3B is the quantitative analysis of the TR. Increasing the voltage from 60 V to 100 V resulted in a significant gain of TR, while a further increase from 100 V to 120 V compromised transfection. Therefore, we took 100 V as the optimized voltages for further studies of HEK-293A cell transfection. Along with the TR, cell viability would also affect the final yield of viable transfected cells. Using PI (propidium iodide) staining and MTT assay, we monitored the cell viability after being transfected (Supplementary Figure S3). The results demonstrated that the transfection process, by either electroporation or Lipo, harmed the cell viability with similar degree. Besides, an excessive voltage, such as 120 V on HEK-293A cells, caused more cell damage than lower voltages did. Since GFP plasmid is relatively small (4.7 Kb), we employed another two larger GFP-tagged plasmids, CaMKII-GFP (7.4 Kb, encoding Calcium/calmodulin Dependent Kinase II) and MG53-GFP (6.2 Kb, encoding Mitsugumin 53), to transfect HEK-293A cells (Supplementary Figure S4). Both CaMKII-GFP and MG53-GFP plasmids expressed efficiently, demonstrating that the FED is capable of transfecting relatively larger plasmids.

One prominent feature of the FED is the ability to rapidly process large amount of cells. We further investigate the relationship between cell flow speed and TR to explore the upper limit of cell processing speed. To ensure every cell experienced 6 electric pulses, the pulse interval was varied according to the speed change (listed in Supplementary Table S4). As shown in Fig. 3C, the TR remained approximately constant as the cell flow rate increased from 0.75 to 4.5 ml/min. A significant drop of TR was observed while the cell flow rate was 5.5 ml/min. Accordingly, the flow velocity was about 2.75 mm/sec, such a velocity produced an shear force (ranged from 1.5 ~ 16.2 μN/cm^2^, calculated by Comsol V3.5a) which is possibly harmful for those electrically stimulated cells, compromising the cell viability, along with the TR. The assessment of the cell viability under different flow rate (Supplementary Figure S3C) also confirmed this point. Therefore, 4.5 ml/min was considered as the maximum cell processing speed of the FED. With the typical cell density 5 × 10^6^ cells/mL, the FED could process 2.25 × 10^7^ cells per minute. To determine the reliability of the microfluidic chip, a prolonged test using HEK-293A cells was performed continuously over the course of 30 minutes. Such time period is enough for the FED to process more than 6 × 10^8^ cells, which equals the total cell number from about 80 culture flasks (75 cm^2^).

**Figure 4:**
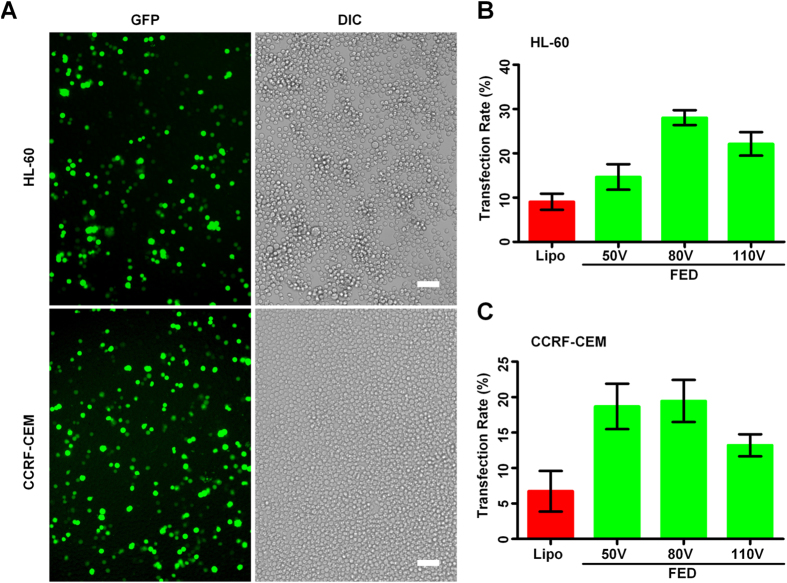
Electrotransfecting HL-60 and CCRF-CEM cells with plasmid DNA. (**A**) The fluorescent images of pEGFP transfected HL-60 and CCRF-CEM cells. The green spots represent transfected cells, which expressed GFP and exhibited green fluorescence. Scale bar 50 μm. Using Lipofectamine2000 as comparison, the transfection rates of HL-60 (**B**) and CCER-CEM (**C**) cells were calculated by dividing the number of GFP-expressing cells by the total cell number. In (**B,C**), the cell flow rate was fixed at 4.5 ml/min. The cell number is counted in five randomly chosen fields of each fluorescent image, using ImageJ from NIH. Each column is the average of three independent assays, each data is showed as the mean ± S.D.

In many practical therapy applications or frontier biological studies, a real challenge is dealing with hard-to-transfected cells, rather than those easy-to transfect cells, such as HEK-293A. To evaluate to capability of FED on transfecting hard-to-transfected cells, we tried two kinds of cells: HL-60, human promyelocytic leukemia cells and CCRF-CEM, a T lymphoblastoid cell line. Both kinds of cells are known to be resistant to chemical transfection approaches such as Lipofectamine 2000. The electroporation voltages for two cell kinds were respectively optimized, and it turned out they had the same optimized voltage, 80 V. As shown in Fig. 4. Compared with Lipo transfection, the FED exhibited a remarkably enhanced TR (HL-60: from less than 10% to about 30%; CCRF-CEM: from 6% to about 20%). In addition, we maintained the high flow speed (4.5 ml/min) in assays for both cell kinds. As comparison, we also evaluated the transfection efficiency and cell viability of a commercial electroporation device (Multiporator^TM^ System, Eppendorf AG, Germany). The results (Supplementary Figure S6) reveal that the FED and the Multiporator^TM^ System show similar transfection efficiency and cell viability. The FED outperformed the commercial devices by its continuous operation mode and corresponding high cell processing speed.

Taken together, DNA transfection assays above reveals that the FED had the ability to electroporate not only easy-to-transfect cell but also hard-to-transfect cells with satisfactory efficiency and cell viability. Meanwhile, the cell processing speed was much higher than previous devices.

### RNA delivery *ex vivo*

Continuously transfecting cultured cells *in vitro* is likely to benefit many laboratorial studies on gene function or drug screening. Meanwhile, recent clinical studies, such as genetically modifying the immune cells to suppress tumor^24^,^25^ and converting erythrocyte into the drug carrier^26^, reveal that continuously transfecting the cells isolated from human blood and transfusing it back to human body for therapeutic purposes is becoming a promising method for clinical therapy. For such *ex vivo* processes, increasing the cell processing speed can accelerate the production of valid therapeutic cells and shorten the exposure duration of vulnerable human cells. Chemical transfection methods are ineffective to most kinds of freshly isolated human cells. As a result, in previous studies, the therapeutic cells were generated by viral-based transfection, which was costly and time-consuming. To determine if the FED can constitute an effective non-viral method for *ex vivo* cell transfection, we use mouse erythrocyte and Cy-5 labelled RNA to mimic a controlled loading and releasing process of gene medicine.

**Figure 5:**
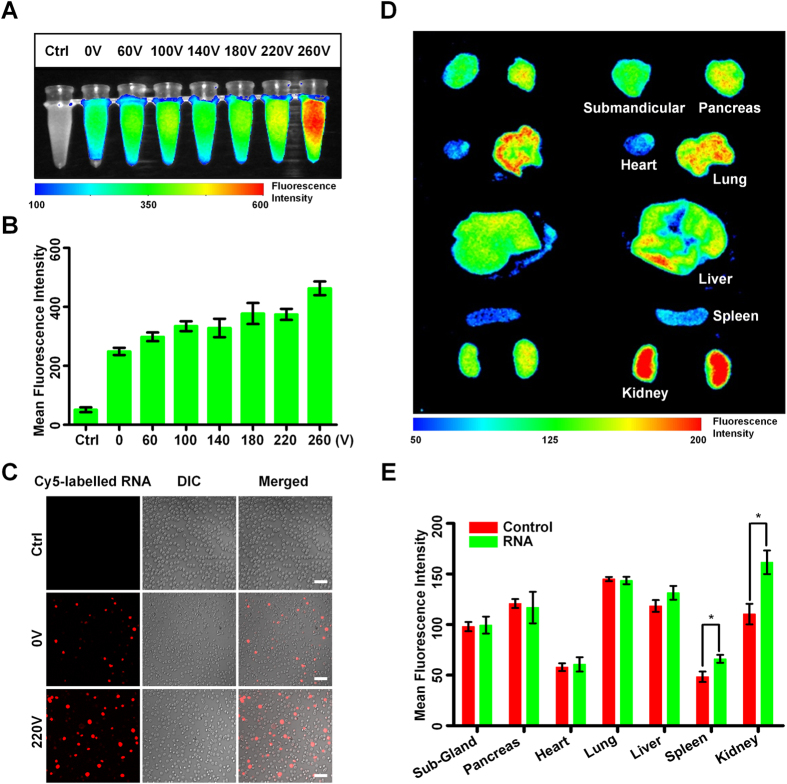
Electroporating mouse erythrocyte as a carrier of RNA. To determine the ex vivo electroporation efficiency of FED, the mouse erythrocytes were freshly isolated and then mixed with electroporation buffer and Cy-5 labelled RNA. The mixtures were electroporated under different voltages using FED. (**A**) The fluorescent image of erythrocytes experienced different electroporation voltages. The most left tube is used as control, loaded by erythrocytes with no RNA added. The second left tube is another kind of control, loaded by erythrocytes mixed with RNA, but experienced no electroporation. (**B**) The quantitative values of the mean fluorescent intensities exhibited in (**A**). (**C**) The con-focal fluorescent images of erythrocytes. The red spots represent erythrocytes loaded with Cy-5 labelled RNA. Scale bar 20 μm. There are barely RNAs remain outside of erythrocytes, revealing the wash process after the electroporation is effective. (**D**) Several internal organs were fluorescently imaged, six hours after re-transfusing the RNA loaded erythrocytes to the mice. Compared to the untreated health mouse (left), the mouse experienced RNA loaded erythrocytes injection (right) exhibited obvious fluorescence in its spleen and kidney, demonstrating a successful releasing process of RNA. The electroporation voltage was 220 V. (**E**) The quantitative values of the mean fluorescent intensities exhibited in (**D**). The red columns represent untreated mice as control, while the green columns represent the mice injected with RNA loaded erythrocytes. *P < 0.1 vs control mice. In (**B**) and (**E**), data is the average of three independent assays and showed as the mean ± S.D.

The detailed experimental protocol is described in section 2.3. Briefly, the mouse erythrocytes were freshly isolated from the blood and mixed with electroporation buffer and RNA solution. The mixture was pumped into FED for electroporation immediately. After RNAs were loaded, the erythrocytes collected from the FED were transfused back into mice as the RNA carrier. Figure 5A is the fluorescent image of the erythrocytes right after being treated by the FED. The erythrocytes experienced no electroporation (0 V) exhibited its ability to load RNA, demonstrated that the *ex vivo* treatment, including washing, purification, pumping and flowing did not harm the functionality of erythrocytes as nucleic acid carrier. As the electroporation voltage was increased from 60 V to 260 V, the fluorescent intensity was remarkably enhanced, revealing the electroporation benefited the RNA loading. The quantitative analysis of the fluorescent intensity (Fig. 5B) further confirmed the RNA loading was proportionally increased with the voltage rising. Compared with unelectroporated erythrocytes, the erythrocytes electroporated by 260 V approximately doubled the RNA loading. To further determine the percentage of RNA-loaded erythrocytes, a con-focal microscopy was used to fluorescently image the electroporated erythrocytes. As shown in Fig. 5C, the electroporation under 220 V efficiently improved the percentage of RNA-loaded erythrocytes, from less than 10% to about 20%. On the other side, the voltage increasing also affected the cell viability. In our assays, excessive voltage (higher than 220 V) would cause severe cell damage, sacrificing the final yield of valid carrier cells. Therefore, 220 V was used for the following studies. We then investigated whether electroporated erythrocytes could effectively unload its cargo to expected target tissue. Six hours after re-transfusing the electroporated erythrocytes, several internal organs were fluorescently imaged to evaluate the released amount of RNA (Fig. 5D). As expected, the RNA-loaded erythrocytes released Cy-5 labelled RNA into kidney and spleen. These two organs were previously found to be the target of erythrocyte-based nucleic acid delivery^27^. The quantitative analysis of the fluorescent intensity (Fig. 5E) further confirmed the electroporation treatment of erythrocytes successfully enhanced the concentration of the RNA in the target tissue. Therefore, a potential application of the FED is to deliver siRNA into such tissues as therapeutic agents. In above *ex vivo* assays, we maintained the high flow rate (4.5 ml/min). From taking blood to re-transfusing treated erythrocytes, the whole *ex vivo* treating process was finished in only 1 hour.

## Conclusion

Continuously electroporation has been demonstrated to be a promising method to genetically transfect massive amounts of cells. The previous macro-scale devices suffered from its high voltage and corresponding adverse effects which compromised both transfection efficiency and cell viability. The applications of the emerging micro-fluidic-based devices were still limited by its low cell processing speed. This study advances this field with a different device in which a large-sized flow tube and a small-spaced needle electrode array were integrated together to ensure high cell processing speed and satisfactory transfection efficiency simultaneously. Compared with previously reported micro-scale or macro-scale continuous electroporation devices, this new device has the following prominent features: i) The large cross-sectional area of the glass tube enables high flow rate, simple flow characterization and low shear force; ii) The specially- arranged electrodes array ensures an even-distributed electric field, along with reduced cell-harming effects. Furthermore, another clear advantage of the device was its simple fabrication process which relieved the requirements for cleanroom facilities. As a result, this device was more accessible and modifiable to potential users than micro-fluidic devices. The efficient nucleic acid transfection was verified both *in vitro* and *ex vivo*. The *in vitro* assays reveal that the DNA transfection efficiencies on three different cells lines, including easy-to-transfect type as well as hard-to-transfect ones, thus are satisfactory to fulfil the demands of biologicals studies. The RNA delivery into freshly isolated erythrocytes demonstrated that the *ex vivo* electroporation by FED successfully enhanced the RNA loading and targeted releasing efficiency of erythrocyte as therapeutical carrier. For both *in vitro* and *ex vivo* electroporation, the FED maintained a high cell processing speed, 2.25 × 10^7^ cells per minute.

Overall, this study realized a fine balance between cell processing speed and cell transfection rate by developing a new method, combining large flow tube and well-organized small electrode array together. The validation of the FED device exhibits the potential of our method for not only laboratory biological studies but also practical therapeutical applications.

## Additional Information

**How to cite this article**: Zhao, D. *et al.* A Flow-Through Cell Electroporation Device for Rapidly and Efficiently Transfecting Massive Amounts of Cells *in vitro* and *ex vivo*. *Sci. Rep.* **6**, 18469; doi: 10.1038/srep18469 (2016).

## Supplementary Material

Supplementary Information
