# Supplementary material for: A Flow-Through Cell Electroporation Device for Rapidly and Efficiently Transfecting Massive Amounts of Cells in vitro and ex vivo
Source: Sci Rep. 2016 Jan 5;6:18469. doi: 10.1038/srep18469 (PMC4700452; doi:10.1038/srep18469)
Supplement: Supplementary Information [file srep18469-s1.doc]

A Flow-Through Cell Electroporation Device for Rapidly and Efficiently Transfecting Massive Amounts of Cells *in vitro* and *ex vivo*

Deyao Zhao^a^, Dong Huang^b^, Yang Li^a^, Mengxi Wu^b,c^, Wenfeng Zhong^b^, Qiang Cheng^a^, Xiaoxia Wang^a^, Yidi Wu^a^, Xiao Zhou^a^, Zewen Wei^d^*, Zhihong Li^b^*, and Zicai Liang^a^*

^a^ Institute of Molecular Medicine, Peking University, Beijing 100871, China

*^b^ National Key Laboratory of Science and Technology on Micro/Nano Fabrication, Institute of Microelectronics, Peking University, Beijing 100871, China*

*^c^* *Department of Engineering Science and Mechanics, The Pennsylvania State University, State College, PA 16801, USA*

*^d^ National Center for Nanoscience and Technology, Beijing 100190, China*

* Authors for correspondence, Zhihong Li (zhhli@ime.pku.edu.cn, Fax 86-10-62751789), Zicai Liang (liangz@pku.edu.cn, Fax +86-10-62769862), or Zewen Wei (weizw@nanoctr.cn, Fax 86-10-82545752).

**Supplementary FigureS1**

*The operation of the flow-through cell electroporation device (FED).*

*
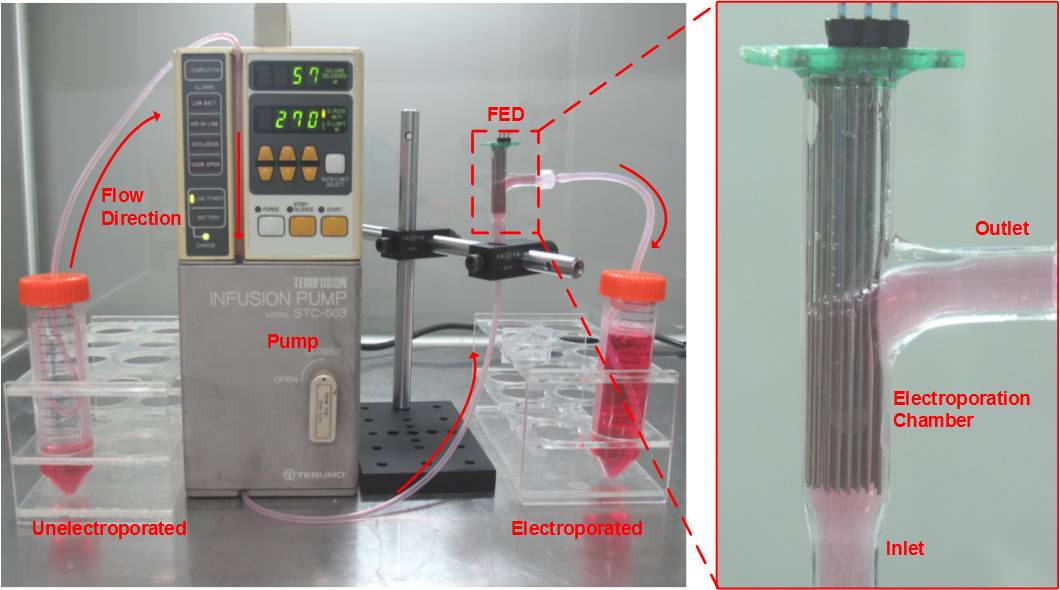
*

To process the flow-through cell electroporation, an infusion pump was used to push the cells into the FED. The syringe pump can also be used while processing relatively small volume of cells. To clearly indicate the cell flow path, the cell culture medium (red fluid) was used in above images. The flow direction was marked by red arrows. The close-up image (right) of FED shows the position of inlet, outlet, electroporation chamber and the upper part of the glass tube which is sealed by PDMS.

**Supplementary Figure S2**

*The fabrication of the flow-through cell electroporation device (FED).*

*
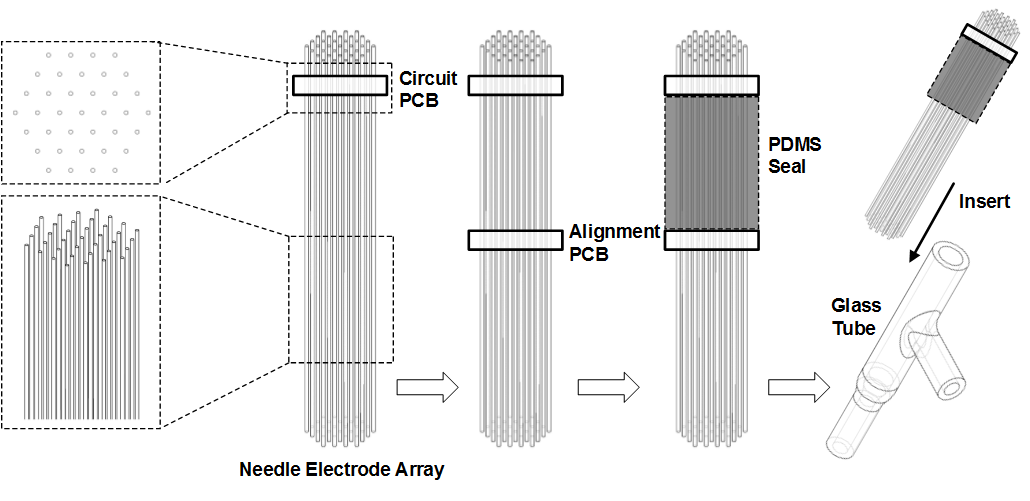
*

The fabrication process of the FED is schemed in above figure. First, 37 commercial stainless steel acupuncture needles were carefully arranged and manually welded on a PCB to from the needle electrode array (NEA). There were 37 bonding pads which were one-to-one corresponded to needles on the PCB. A switch circuit was designed on the PCB to shift the electrical connection. Second, to prevent the NEA form short-circuit, another alignment PCB was assembled on the middle area of the NEA. The alignment PCB has no electrical circuit but 37 via holes which are one-to-one corresponded with bonding pads on circuit PCB. By threading every needle through its respective via holes, all needles maintained approximately parallel. Third, using the circuit PCB and alignment PCB as the boarder, proper amount of PDMS was cured between two PCBs to electrically isolate the circuit PCB from the cell flow. Also the needles between two PCBs were isolated from each other. Finally, after extracting the alignment PCB, the NEA was inserted into the glass tube through the top opening to finish the fabrication of FED.

**Supplementary Figure S3**

*The cell viability determination*

**
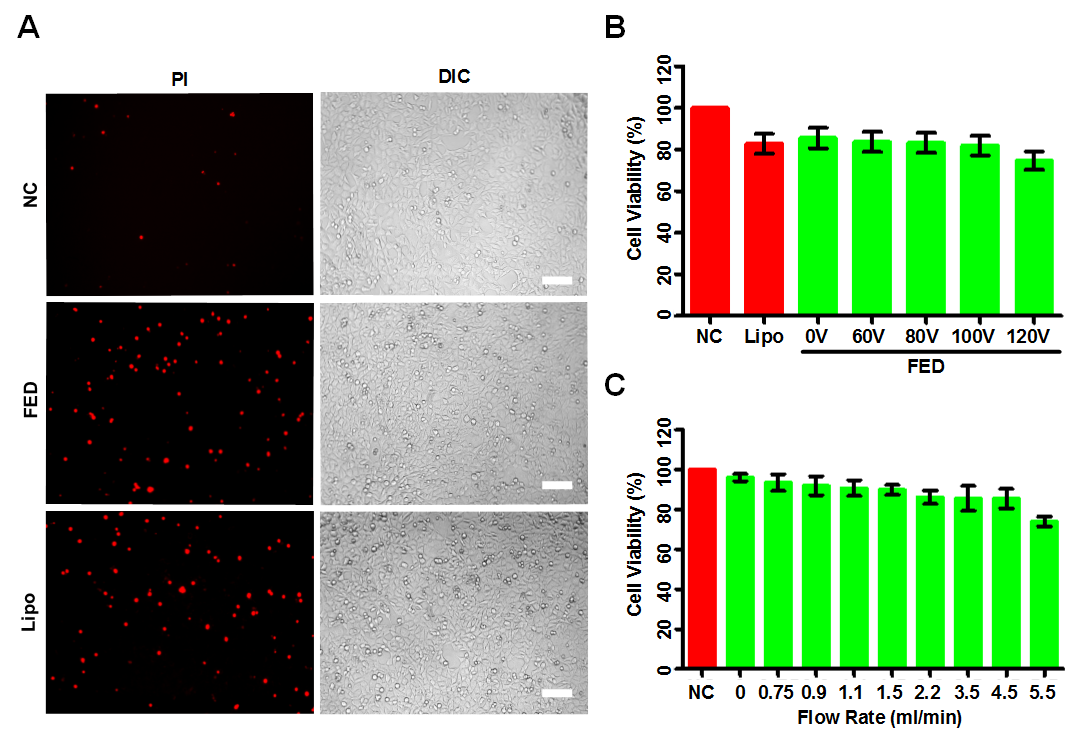
**

**(A) The PI staining of the cells transfected by FED and Lipo, respectively.**

HEK-293A cells and PI (propidium iodide) staining were used to visually monitoring the cell viability. As shown in the fluorescent images, the membrane of fatally damaged cell became permanently permeable, being stained to red by PI. Therefore the red spots represented the dead cells. Using the cells which experienced neither FED nor Lipo transfection (top) as the control, both FED (middle) and Lipo (bottom) transfection compromised the cell viability with a similar degree. Scale bar 100 μm. The cells were fluorescently imaged 24 hours after transfection. The voltage used in FED electroporation is 100 V. The Lipofectem2000 was purchased from Invitrogen, and the cell transfection was finished following the product instruction.

**(B) The relationship between cell viability and electroporation voltage.**

To further quantitatively determine the cell viability, MTT assays was employed. Briefly, the electroporated cells were collected and cultured in the 96-well plate. 24 hours after electroporation, cell culture medium was replaced by 100 mL fresh complete DMEM and 2 μL MTT (5 μg/μL) each well. All medium was removed 4 hours later, followed by adding 50 μL DMSO and incubating for 10 minutes at 37 ℃. Finally, the absorbance read at 540 nm with a reference wavelength of 650 nm. The net absorbance OD_net540_ was OD_540_ minus OD_650_. Cell viability was calculated as:

Cell Viability (%) = (OD_net540(sample)_ / OD_net540(mock)_) X 100%

As the voltage was enhanced from 0 to 120 V, the cell viability was gently reduced. While the voltage was increased to 120 V, the FED electroporation induced more cell damage than Lipo transfection did. Each column is the average of three independent assays, each data is showed as the mean ± S.D..

**(C) The relationship between cell viability and cell flow rate**

Using the same MTT method, the relationship between cell viability and flow rate was determined. The pulse interval was adjusted according to the flow rate (listed in Supplementary Table S4) to ensure every cell experiences the same 6 electrical pulses. As the flow rate was increased from 0 to 4.5 ml/min, the cell viability was mildly reduced. While 5.5 ml/min was used, the cell viability was obviously harmed. This gave us a proper range of cell rate. Each column is the average of three independent assays, each data is showed as the mean ± S.D..

**Supplementary Figure S4**

*Transfecting HEK-293A cells with pEGFP-C3, CaMKII-GFP and MG53-GFP plasmids*


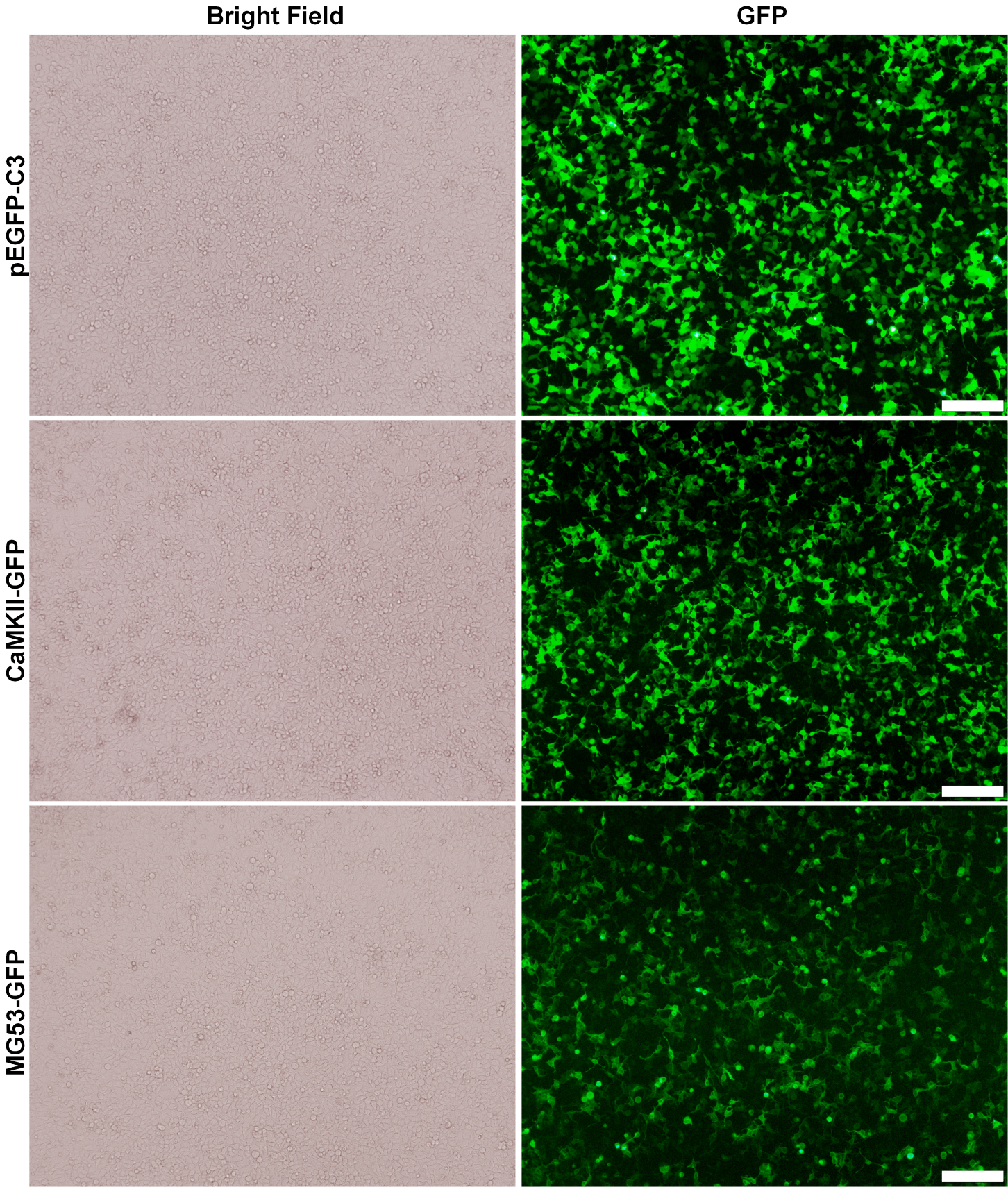


Considering pEGFP-C3 is a relatively small plasmid (4.7 Kb), we employed another two larger plasmids, CaMKII-GFP (7.4 Kb) and MG53-GFP (6.2 Kb), to further demonstrate the capability of FED to transfect larger plasmids. In both CaMKII-GFP and MG53-GFP plasmid, GFP was used a tracing tag to indicate the expression of functional protein, respectively CaMKII (Calcium/calmodulin dependent kinase II) and MG53 (Mitsugumin 53). For all three kinds of plasmid, we used identical protocols and parameters described in Experimental Section. The fluorescent images reveal that the HEK-293A cells expressed pEGFP-C3 and CaMKII-GFP plasmids with approximately the same efficiency, while the transfection efficiency of MG53-GFP is slightly lower. Scale bar 200 μm**.**

**Supplementary Table S5**

*The one-to-one correspondences between cell flow velocity and electrical pulse interval.*

| Flow Velocity (ml/min) | 0.75 | 0.9 | 1.1 | 1.5 | 2.2 | 3.5 | 4.5 | 5.5 |
| --- | --- | --- | --- | --- | --- | --- | --- | --- |
| Pulse Interval (s) | 6.6 | 5.5 | 4.4 | 3.3 | 2.2 | 1.4 | 1.1 | 0.9 |

While exploring the optimum flow velocity, to ensure every cell experienced 6 electrical pulses, the pulse interval was reduced as the flow velocity was increased. The one-to-one correspondences are listed above.

**Supplementary Figure S6**

*Electroporation of HL-60 cells by FED and Eppendorf Multiporater^TM^ System*

**
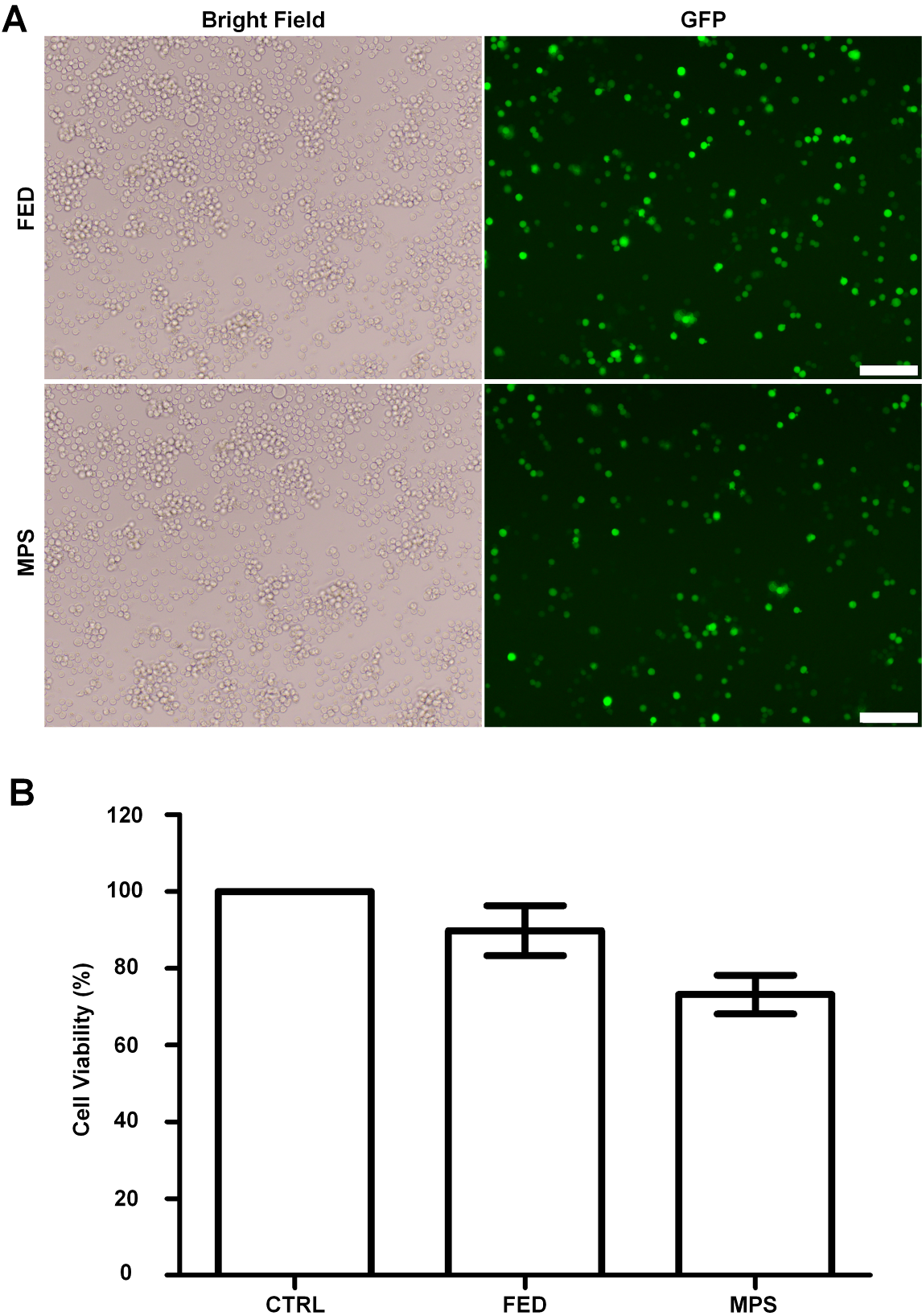
**

To compare the performance of FED and a commercial electroporation device (Multiporator^TM^ System, Eppendorf AG, Germany, Hereinafter referred to MPS). We transfected HL-60 cells with GFP plasmid. We also evaluated the cell viability by MTT assays. The FED electroporation follows the same protocol described in Experimental Section. To perform MPS electroporation, HL-60 cells were firstly harvested and resuspended to a density of about 7.5×10^6^ cells/mL in the hypo-osmolar buffer (25 mM KCl, 0.3 mM KH_2_PO_4_, 0.85 mM K_2_HPO_4_, 36 mM myo-inositol), then pEGFP-C3 plasmid was added to a final concentration of 20 μg/mL. According to the operation instruction of MPS, we employed a trail-and-error process to acquire the optimum electroporation voltage, which was found to be 250 V. This voltage is about three times as high as the voltage used in FED (80 V). Following the instruction of MPS, it took us about 3 minute (including cell loading/unloading and cuvette docking/releasing; not including parameter adjusting and cuvette washing) to electroporate 5×10^5^ cells. As comparison, the FED processes about 2×10^7^ cells per minute.

1. **The GFP plasmid transfection efficiency**

The fluorescent images of electroporated HL-60 cells reveal that FED and MPS exhibited similar transfection rate, around 30%. Scale bar 100 μm

**(B) The cell viability evaluated by MTT assay**

MTT assays were introduced to evaluate the cell viability. Electroporated HL-60 cells were cultured for 24 hours in a 96-well plate, the following protocols is the same as describe in the figure legend of Supplementary Figure S3B. Each column is the average of three independent assays, each data is showed as the mean ± S.D.. Compared with control group in which normally cultured cells experienced no treatment, FED compromised cell vitality to 89%, while MPS was with an even lower viability 73%. The reason for this difference is probably because FED utilizes lower voltage (80 V vs 250 V) and making the cell solution continuously flow, therefore alleviating the accumulation of harmful effects, including excessive heat, pH value change, even bubbles generated form water electrolysis.
